# Mitochondrial Contributions in the Genesis of Delayed Afterdepolarizations in Ventricular Myocytes

**DOI:** 10.3389/fphys.2021.744023

**Published:** 2021-10-14

**Authors:** Vikas Pandey, Lai-Hua Xie, Zhilin Qu, Zhen Song

**Affiliations:** ^1^Department of Medicine, David Geffen School of Medicine, University of California, Los Angeles, Los Angeles, CA, United States; ^2^Department of Cell Biology and Molecular Medicine, Rutgers New Jersey Medical School, Newark, NJ, United States; ^3^Department of Computational Medicine, David Geffen School of Medicine, University of California, Los Angeles, Los Angeles, CA, United States; ^4^Peng Cheng Laboratory, Shenzhen, China

**Keywords:** delayed afterdepolarization, Ca^2+^ wave, mitochondrion, cardiac cell, Ca^2+^ signaling

## Abstract

Mitochondria fulfill the cell’s energy demand and affect the intracellular calcium (Ca^2+^) dynamics *via* direct Ca^2+^ exchange, the redox effect of reactive oxygen species (ROS) on Ca^2+^ handling proteins, and other signaling pathways. Recent experimental evidence indicates that mitochondrial depolarization promotes arrhythmogenic delayed afterdepolarizations (DADs) in cardiac myocytes. However, the nonlinear interactions among the Ca^2+^ signaling pathways, ROS, and oxidized Ca^2+^/calmodulin-dependent protein kinase II (CaMKII) pathways make it difficult to reveal the mechanisms. Here, we use a recently developed spatiotemporal ventricular myocyte computer model, which consists of a 3-dimensional network of Ca^2+^ release units (CRUs) intertwined with mitochondria and integrates mitochondrial Ca^2+^ signaling and other complex signaling pathways, to study the mitochondrial regulation of DADs. With a systematic investigation of the synergistic or competing factors that affect the occurrence of Ca^2+^ waves and DADs during mitochondrial depolarization, we find that the direct redox effect of ROS on ryanodine receptors (RyRs) plays a critical role in promoting Ca^2+^ waves and DADs under the acute effect of mitochondrial depolarization. Furthermore, the upregulation of mitochondrial Ca^2+^ uniporter can promote DADs through Ca^2+^-dependent opening of mitochondrial permeability transition pores (mPTPs). Also, due to much slower dynamics than Ca^2+^ cycling and ROS, oxidized CaMKII activation and the cytosolic ATP do not appear to significantly impact the genesis of DADs during the acute phase of mitochondrial depolarization. However, under chronic conditions, ATP depletion suppresses and enhanced CaMKII activation promotes Ca^2+^ waves and DADs.

## Introduction

Delayed afterdepolarizations (DADs) are abnormal depolarizations during the diastolic phase following an action potential (AP) and could trigger cardiac arrhythmias (Rosen et al., 1984; January and Fozzard, 1988; Katra and Laurita, 2005; Qu et al., 2014; Song et al., 2017). DADs are known to be caused by spontaneous calcium (Ca^2+^) waves (Rosen et al., 1984; Marban et al., 1986; January and Fozzard, 1988), occurring due to spontaneous Ca^2+^ release from the intracellular Ca^2+^ store, sarcoplasmic reticulum (SR), *via* the ryanodine receptors (RyRs). Ca^2+^ waves are known to be promoted by Ca^2+^ overload under normal (Cheng et al., 1996) and pathological conditions, such as heart failure (Pogwizd and Bers, 2003; Hoeker et al., 2009), long QT syndrome (Mohler et al., 2003), ischemia (Ross and Howlett, 2009), and catecholaminergic polymorphic ventricular tachycardia (CPVT) (Watanabe et al., 2009). During a cardiac cycle, Ca^2+^ enters into the cytosol from the extracellular space mainly *via* L-type Ca^2+^ channels (LCCs) during membrane depolarization, which causes Ca^2+^ release from the SR, a process called Ca^2+^-induced Ca^2+^ release (CICR; Bers, 2002). Ca^2+^ is extruded from the cell mainly through the Na^+^-Ca^2+^ exchanger (NCX) and taken back to the SR through sarcoplasmic reticulum Ca^2+^-ATPase (SERCA). Meanwhile, mitochondria, as another Ca^2+^ store, are involved in intracellular Ca^2+^ cycling *via* mitochondrial Ca^2+^ uniporter (MCU; Baughman et al., 2011; De Stefani et al., 2011), mitochondrial Na^+^-Ca^2+^ exchangers (mNCX; Palty et al., 2010), and the mitochondrial permeability transition pore (mPTP; Hunter et al., 1976). Besides the direct Ca^2+^ exchange, mitochondria may indirectly alter the cytosolic Ca^2+^ dynamics through many different ways under abnormal conditions (Yan et al., 2008; Florea and Blatter, 2010; Zhao et al., 2013; Xie et al., 2018), thus impacting Ca^2+^ waves and DADs. Under normal conditions, the occurrence of mitochondrial depolarizations through the mPTP opening is rare (Lu et al., 2016). However, the mPTP open probability increases in abnormal conditions, resulting in a higher degree of mitochondrial depolarization in the cell. The critical consequences that affect intracellular Ca^2+^ dynamics include an increased cytosolic reactive oxygen species (ROS) level, enhanced Ca^2+^/calmodulin-dependent protein kinase II (CaMKII) activation *via* oxidative stress, Ca^2+^ influx into the cytosol from the mitochondria, and a decrease in the cytosolic ATP level, etc.

Reactive oxygen species can directly affect the RyRs hyperactivity and SERCA pump strength *via* its redox effect (Zima and Blatter, 2006; Wagner et al., 2013) or indirectly *via* oxidized CaMKII signaling (Xie et al., 2009; Foteinou et al., 2015). CaMKII activation is known to increase SERCA pump through phosphorylation of phospholamban (Hund and Rudy, 2004; Mattiazzi and Kranias, 2014), make RyRs leakier (Ai et al., 2005), and modulate LCCs and other membrane ionic currents (Anderson et al., 1994; Xiao et al., 1994; Yuan and Bers, 1994; Hund and Rudy, 2004; Hund et al., 2008). Furthermore, ATP depletion impairs the SERCA pump function (Sakamoto and Tonomura, 1980). Due to their highly complex interactions, it is difficult to dissect out the individual roles of mitochondrial Ca^2+^ exchange, ROS, ATP, and CaMKII activation in the genesis of DADs during mitochondrial depolarization by experiments. We have recently developed a whole-cell ventricular myocyte model consisting of a network of intermingled Ca^2+^ release units (CRUs) and mitochondria, which contains physiological details of mitochondrial membrane potential, mitochondrial Ca^2+^ cycling, mPTP stochastic opening and closing, intracellular ROS, and oxidized CaMKII signaling. Using this model, we have investigated the underlying mechanisms of Ca^2+^ alternans and early afterdepolarizations caused by mitochondrial depolarization and dissected each of the components (Xie et al., 2018; Song et al., 2019; Pandey et al., 2021).

We used this model to investigate the underlying mechanisms of spontaneous Ca^2+^ release mediated DADs under mitochondrial depolarization due to mPTP openings in the present work. Specifically, we performed computer simulations to reveal individual contributions of the components mentioned earlier to the genesis of Ca^2+^ waves and DADs. Our previous work provided mechanistic insights of generation of Ca^2+^ alternans under mitochondrial depolarization, and we have reported that the redox effect of ROS on RyRs and SERCA pump synergistically promote alternans (Pandey et al., 2021). Here, we show that the ROS redox regulation of RyRs plays a significant role in the genesis of Ca^2+^ waves and DADs during the acute phase of mitochondrial depolarization. Also, upregulation of MCU can promote DADs through Ca^2+^-dependent openings of mPTPs. However, the changes of oxidized CaMKIIs activation and the cytosolic ATP level are at much slower time scales than the redox effects of ROS, and thereby, they do not significantly impact the genesis of DADs in a relatively short duration after mitochondrial depolarization. Whereas, under chronic conditions, ATP depletion suppresses and enhanced CaMKII activation promotes the Ca^2+^ waves and DADs.

## Materials and Methods

The details of the model, including the mathematical formulations and control values of the parameters, can be found in Song et al. (2019) and Pandey et al. (2021). Here, we describe some of the essential aspects of the model for the sake of this study.

### The Overall Ventricular Myocyte Model Structure

Our rabbit ventricular myocyte model consists of a 3-dimensional coupled network of CRUs and mitochondria. This network contains 21504 (64 2812) CRUs and 5376 (64 14 6) mitochondria. The membrane potential (V) of the cell is described by


(1) $$C_{m}\,\frac{d\,V}{d\,t}=I_{N\,a}+I_{N\,a,L}+I_{C\,a,L}+I_{N\,C\,X}+I_{K\,1}+I_{K\,r}+I_{K\,s}+I_{t\,o,f}+I_{t\,o,s}+I_{N\,a\,K}+I_{K,A\,T\,P}+I_{C\,a,b}-I_{s\,t\,i}$$

where *C*_*m*_=1 mF/cm^2^ is the cell membrane capacitance, and *I*_*sti*_ is the stimulus pulse with the current density being −80 mA/cm^2^ and the duration being 0.5 ms.

### Regulations of Reactive Oxygen Species and CaMKII on Ryanodine Receptors

The oxidized CaMKII activation and the redox effect of ROS both increase the open probability of RyRs (Wehrens et al., 2004; Ai et al., 2005; Guo et al., 2006; Zima and Blatter, 2006; Wagner et al., 2013). To incorporate these effects, the close-to-open rate (*k*_*12*_) of RyRs was modeled as follows:


(2) $$k_{12}=k_{b\,a\,s\,e}\,k_{u}\,\left(1+△\,k_{C\,a\,M\,K\,I\,I}+△\,k_{R\,O\,S}\right)\,{\left({\left[C\,a^{2+}\right]}_{p}\right)}^{2}$$

(3) $$△\,k_{C\,a\,M\,K\,I\,I}=\frac{\Delta \,k_{C\,a\,M\,k,m\,a\,x}}{1+{\left(\frac{\Delta \,k_{C\,a\,M\,R\,y\,R}}{{\left[C\,a\,m\,K\,I\,I\right]}_{a\,c\,t}}\right)}^{h_{C\,a\,M\,K\,I\,I\,y\,R}}}$$

(4) $$△\,k_{R\,O\,S}=\frac{\Delta \,K_{R\,O\,S,m\,a\,x}}{1+{\left(\frac{\Delta \,k_{m\,R\,O\,S\,R\,y\,R}}{{\left[R\,O\,S\right]}_{c\,y\,t}}\right)}^{h_{R\,O\,S\,R\,y\,R}}}$$

where △*k*_*CaMKII*_ and △*k*_*ROS*_ are the CaMKII-dependent (Eq. 3) and ROS-dependent components (Eq. 4), respectively. *k*_*base*_ and *k_u_* are the rate constants. [*Ca*^2 +^ ]_*p*_ is the Ca^2+^ concentration in the dyadic space of a CRU. [*CaMKII*]_*act*_ and [*ROS*]_*cyt*_ are the CaMKII activation level and the cytosolic ROS concentration in each CRU, respectively. The increase in CaMKII activation and ROS level increase *k*_*12*_, which in turn increases the open probability of RyRs.

### Regulations of Reactive Oxygen Species and CaMKII on Sarcoplasmic Reticulum Ca^2+^-ATPase Pump

The formulation of SERCA is


(5) $${\text{J}}_{\text{up}}=v_{u\,p}\,f_{u\,p,A\,T\,P}\,f_{u\,p,R\,O\,S}\,\frac{{\left[C\,a^{2+}\right]}_{i}^{2}}{{\left[C\,a^{2+}\right]}_{i}^{2}+{\left(K_{i}-P\,L\,B\,\left({\left[C\,a\,M\,K\,I\,I\right]}_{a\,c\,t}\right)\right)}^{2}}$$

where *f*_*up,ATP*_, and *f*_*up,ROS*_ are ATP and ROS-dependent functions (Song et al., 2019):


(6) $$f_{u\,p,A\,T\,P}=\frac{1}{1+\frac{{\left[A\,D\,P\right]}_{f}}{k_{i,u\,p}^{\prime }}+\left(1+\frac{{\left[A\,D\,P\right]}_{f}}{k_{i,u\,p}}\right)\,\frac{k_{m\,u\,p}\,A\,T\,P}{\left[A\,T\,P\right]}}$$

(7) $$f_{u\,p,R\,O\,S}=\frac{1}{1+{\left(\frac{{\left[R\,O\,S\right]}_{c\,y\,t}}{k_{d,r\,o\,s}}\right)}^{h_{r\,o\,s,S\,E\,R\,C\,A}}}+\frac{0.75}{1+{\left(\frac{k_{d,r\,o\,s}}{{\left[R\,O\,S\right]}_{c\,y\,t}}\right)}^{h_{r\,o\,s,S\,E\,R\,C\,A}}}$$

*v*_*up*_ is the maximum SERCA strength and *K*_*i*_the half-maximum value. *PLB*([*CaMKII*]_*act*_) is a CaMKII dependent function. [*CaMKII*]_*act*_ is CaMKII activation level in the cytosolic space of a CRU.

### The Mitochondrial Permeability Transition Pore Model

We used a 3-state (two close states C_0_ and C_1_, and an open state O) Markov model to simulate the stochastic opening and closing of the mPTP. The transition rate from the C_0_ state to the C_1_ state, *k*_*c0c1*_, is set as:


(8) $$k_{c\,0\,c\,1}=\alpha_{0}\,\left(1+199*\frac{{\left[C\,a^{2+}\right]}_{m}^{h_{m\,P\,T\,P}}}{{\left[C\,a^{2+}\right]}_{m}^{h_{m\,P\,T\,P}}+{\left[C\,a^{2+}\right]}_{0}^{h_{m\,P\,T\,P}}}\right)$$

where *h*_*mPTP*_ is the Hill coefficient, [*Ca*^2 +^]_*m*_ is the mitochondrial free Ca^2+^ in the corresponding mitochondrion, and [*Ca*^2 +^ ]_0_ is the half-maximum value. We assume that other transition rates are constants. To simulate different levels of mPTP open probability, we multiplied a factor, α_*mPTP*_, to the transition rate from C_1_ to O, $k_{c\,10}^{0}$, i.e.,


(9) $$k_{c\,1\,o}=\alpha_{m\,P\,T\,P}\,k_{c\,1\,o}^{0}$$

## Results

### Mitochondrial Depolarization Due to Openings of Mitochondrial Permeability Transition Pore Promotes Spontaneous Ca^2+^ Release and Delayed Afterdepolarizations

**FIGURE 1:**
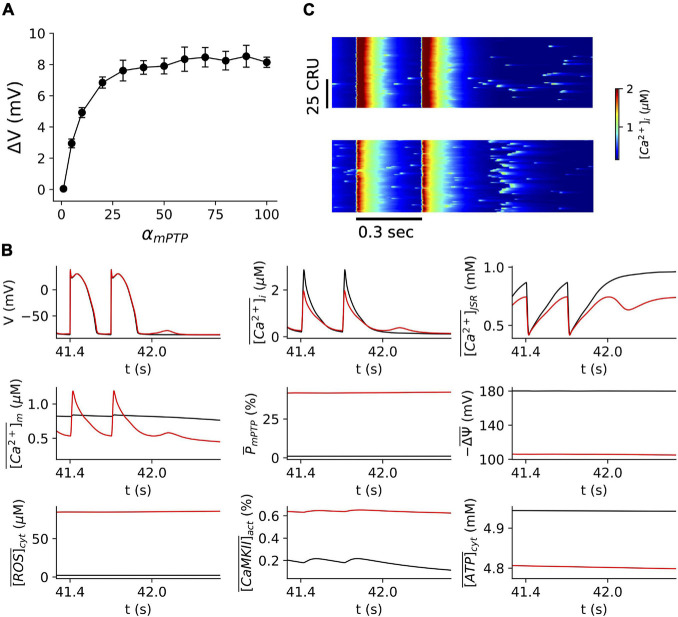
Mitochondrial depolarization *via* mPTP opening promotes DADs. **(A)** The amplitude of DADs vs. α_*mPTP*_. The resting potential is –86.0 mV. Note that for the range of α_*mPTP*_ in these simulations, we observed only one DAD after stopping pacing. **(B)** Time traces of V, ${\bar{\left[C\,a^{2+}\right]}}_{i}$, ${\bar{\left[C\,a^{2+}\right]}}_{J\,S\,R}$, ${\bar{\left[C\,a^{2+}\right]}}_{m}$, ${\bar{P}}_{m\,P\,T\,P}$, $-\bar{△\,\psi }$, ${\bar{\left[R\,O\,S\right]}}_{c\,y\,t}$,${\bar{\left[C\,a\,M\,K\,I\,I\right]}}_{a\,c\,t}$, ${\bar{\left[A\,T\,P\right]}}_{c\,y\,t}$ for normal control (α_*mPTP*_=1) in black and mitochondrial depolarization conditions (α_*mPTP*_=60) in red, respectively. The pacing cycle length is 300 ms, and we stopped pacing after 140 beats (i.e., 42 s). This pacing protocol was used throughout the whole study. **(C)** Linescan images of the cytosolic Ca^2+^ for normal (top) and mitochondrial depolarization (bottom) conditions as in panel **(B)**.

We investigated the impact of mitochondrial depolarizations on the occurrence of Ca^2+^ waves and DADs *via* mPTP opening. We performed simulations over a wide range of α_*mPTP*_ values at the PCL of 300 ms (Figure 1A). α_*mPTP*_ is a factor multiplied to transition rate of mPTP opening, and increasing its value results in higher mPTP opening. For each simulation, the cell was paced for 140 beats (42 s), following 3 s without pacing in order to observe Ca^2+^ waves and DADs. As shown in Figure 1A, the amplitude of DAD increases with α_*mPTP*_, suggesting that mitochondrial depolarization due to openings of mPTP promotes spontaneous Ca^2+^ release and DADs. Also, when α_*mPTP*_ is greater than ∼50, the proarrhythmic effect appears to saturate. Under the control condition (α_*mPTP*_=1), there is no occurrence of DADs (Figures 1A,B). The cytosolic ROS is ∼2 μM, CaMKII activation is ∼0.2%, the cytosolic ATP is ∼5 mM, and most of the mitochondria remain repolarized ($-\bar{\Delta \,\psi }=$∼180 mV, and the mPTP open probability ∼0.8%, mitochondrial Ca^2+^ amplitude is ∼0.8 μM) (Figure 1B). However, with the higher open probability of mPTP (∼42%, for α_*mPTP*_=60), we observed DADs. The corresponding line-scan image clearly shows enhanced spontaneous Ca^2+^ release as compared to a few scattered Ca^2+^ sparks under the control condition (Figure 1C). In this case, we should note that the mitochondrial Ca^2+^ amplitude increased to ∼1.2 M, and the cytosolic ROS drastically increased to ∼86 μM. Still, the CaMKII activation and the cytosolic ATP level insignificantly changed (∼0.6% and ∼4.8 mM, respectively).

### Role of Reactive Oxygen Species in the Genesis of Delayed Afterdepolarizations

**FIGURE 2:**
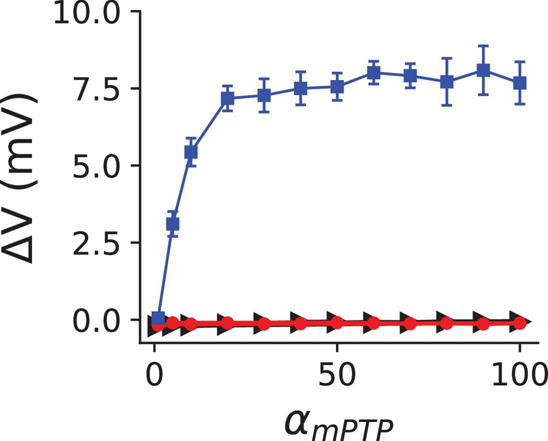
The Redox effect of cytosolic ROS on RyR facilitates the genesis of DADs. The mean and standard deviation of the DAD amplitude vs. α_*mPTP*_ for three different conditions: clamped cytosolic ROS level at 1.0 μM (red circles), the redox effect of ROS only exerted on RyRs (blue squares), and the redox effect of ROS only exerted on SERCA (black triangles). The removal of the redox effect of ROS on RyRs and SERCA was executed by setting △*k*_*ROS*_=0 in Eq. 2, and *f*_*up*,*ROS*_=1 in Eq. 5, respectively. Ten random trials were performed for each given α_*mPTP*_ value.

The concentration of the cytosolic ROS depends on the level of mitochondrial depolarization, and thus, increasing α_*mPTP*_ increases the open probability of mPTP, which, in turn, elevates the level of cytosolic ROS. To further identify the role of cytosolic ROS in the genesis of DADs, we performed simulations for a clamped ROS level at the PCL 300 ms. In the free-running ROS case as shown in Figure 1, the ROS dynamics in the model remains intact, while in the clamped ROS condition, the cytosolic ROS is clamped to a constant regardless the level of mitochondrial depolarization is. Here we clamped ROS to be 1.0 μM, which is close to the level under the normal control condition. We then measured the amplitude of DAD with different α_*mPTP*_ values for the clamped ROS (Figure 2, red) condition. We observed that when the ROS was clamped at the control level (1.0 μM), no DADs occurred, suggesting that the cytosolic ROS plays a critical role in inducing DADs during mitochondrial depolarization.

Furthermore, the cytosolic ROS is known to alter the characteristics of both SERCA and RyRs (Zima and Blatter, 2006; Wagner et al., 2013). Therefore, we investigated the redox effect of ROS on the RyRs and SERCA pump separately to dissect out its individual role. We observed that DADs disappeared when we removed the redox effect of ROS on the RyRs (Figure 2, green). However, removing the redox effect on SERCA did not significantly impact the amplitude of DADs (Figure 2, blue), suggesting that the redox effect on SERCA may not play a critical role in the genesis of DADs. In fact, the direct redox effect of ROS inhibits the SERCA pump activity. Thus, removing the redox effect on SERCA increased the SR Ca^2+^ load instead, causing higher amplitudes of DADs. For instance, at α_*mPTP*_=20, the amplitude of DAD is 7.17 mV when the redox effect of ROS is only exerted on RyRs (Figure 2, blue), but 6.84 mV under control as shown in Figure 1A.

### Role of the Mitochondrial Ca^2+^ in the Genesis of the Delayed Afterdepolarizations

Several studies have shown that under certain pathological conditions, MCU activity is enhanced (Santulli et al., 2015; Xie et al., 2018), which may elevate the mitochondrial free Ca^2+^. Our previous study (Song et al., 2019) showed that the increase of MCU up to 20-fold does not alter cytosolic Ca^2+^ markedly at the steady-state. However, we hypothesize that the higher mitochondrial Ca^2+^ due to MCU overexpression could increase the mPTP open probability and cause higher ROS production in the cytosol (Korge et al., 2011). To test this hypothesis, we performed simulations to examine the effect of MCU overexpression on the genesis of DADs.

**FIGURE 3:**
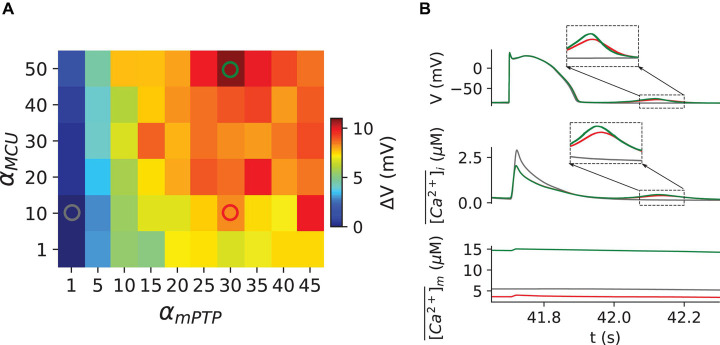
Mitochondrial Ca^2+^ Uniporter upregulation enhances the genesis of DADs through Ca^2+^- dependent opening of mPTP. **(A)** Dependence of the DAD amplitude on α_*MCU*_ and α_*mPTP*_. **(B)** Time traces of V, ${\bar{\left[C\,a^{2+}\right]}}_{i}$, and ${\bar{\left[C\,a^{2+}\right]}}_{m}$ for the (α_*mPTP*_, α_*MCU*_) coordinates as marked in panel **(A)**, following the same color codes. The zoomed-in sections of DAD and spontaneous Ca^2+^ oscillation are shown in the corresponding insets.

We multiplied a factor, denoted as α_*MCU*_, to the maximal MCU conductance. α_*MCU*_=1 represents the control case and higher α_*MCU*_ values are used to represent the different levels of MCU activity. We plotted in Figure 3A the amplitude of DADs for different α_*MCU*_ and α_*mPTP*_. We observed that at α_*mPTP*_ = 1, increasing the MCU activity did not result in DADs even for α_*MCU*_=50. When α_*mPTP*_ becomes greater, the effect of MCU on promoting DADs appears to be more significant. Time traces of membrane voltage, the whole-cell averaged cytosolic Ca^2+^ and the mitochondrial free Ca^2+^ for the three marked locations in the phase map (Figure 3A) are shown in Figure 3B. These results indicate that increasing MCU activity could promote spontaneous Ca^2+^ release and DADs. The mechanism revealed in the model is that increasing MCU activity elevates the mitochondrial free Ca^2+^, which increases the open probability of mPTP, resulting in the elevation of the cytosolic ROS, which in turn promotes the spontaneous Ca^2+^ release and DADs.

### Role of Oxidized CaMKII Activation and ATP in the Genesis of Delayed Afterdepolarizations

As seen in Figure 1B, the CaMKII activation and ATP appeared to change slowly during the simulations due to the slow kinetics in the governing equations of their dynamics. It is computationally cumbersome to run long simulations (up to several thousand beats) for these variables to reach their steady states. Therefore, to evaluate the individual role of CaMKII activation and ATP in the genesis of DADs, we clamped them to different constant values, respectively.

**FIGURE 4:**
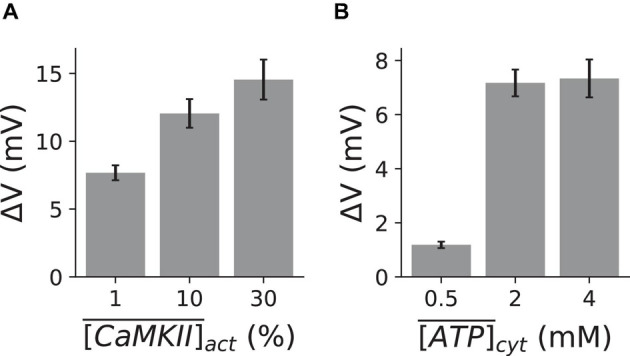
Effect of CaMKII activation and ATP depletion on the genesis of DADs. The mean and standard deviation of the DAD amplitude under three clamped CaMKII activation **(A)** and ATP **(B)** levels, respectively. α_*mPTP*_=30. Ten random trials were performed for each clamped CaMKII activation or ATP level.

Figure 4A shows the relationship between the amplitude of DADs and the CaMKII activation level. Our results clearly show that increasing CaMKII activation promotes DADs. However, due to its slow kinetics, CaMKII activation insignificantly changes during the acute phase of mitochondrial depolarization (Figure 1B), suggesting that CaMKII activation may not play a primary role in the genesis of DADs during the short period immediately after mitochondrial depolarization. Still, it may promote DADs chronically due to its regulation on SERCA, LCC, and RyRs (Wang et al., 2020).

Since the SERCA pump requires ATP for its normal function, a lower level of ATP directly impairs the SERCA pump activity (Eq. 5). However, ATP depletion is a slow process, which is evident from Figure 1B. Hence, we clamped ATP at different levels for α_*mPTP*_ = 30 from the beginning of the simulations. Figure 4B plots the relationship between the amplitude of DADs and the cytosolic ATP concentration, and it shows that ATP depletion suppresses DADs during mitochondrial depolarization (α_*mPTP*_ = 30). Our results demonstrate that the cytosolic ATP level has a significant impact on the genesis of DADs. However, the depletion of the cytosolic ATP concentration during mitochondrial depolarization is a relatively slow process. Therefore, ATP depletion should not play a central role in the genesis of DADs during the acute phase of mitochondrial depolarization. Thus, similar to CaMKII activation, ATP depletion may only suppress DADs in a much longer time scale.

## Discussion

We used a physiological detailed ventricular myocyte model consisting of a 3D network of coupled CRUs and mitochondria to investigate the roles of mitochondrial depolarization *via* mPTP opening in the genesis of DADs. The systematic investigation of individual roles, including the cytosolic ROS, mitochondrial Ca^2+^, CaMKII activation, and the cytosolic ATP, reveals that the redox effect of ROS on RyRs may play an essential role in the occurrence of DADs during the acute phase of mitochondrial depolarization. Furthermore, increasing the MCU activity could promote DADs by increasing the mPTP open probability through mitochondrial Ca^2+^ dependent kinetics of mPTP. In addition, oxidized CaMKII activation promotes, and ATP depletion suppresses DADs chronically in the condition of mitochondrial depolarization.

### Role of Reactive Oxygen Species, CaMKII Activation and ATP Depletion on the Genesis of Delayed Afterdepolarizations

Experimental findings reported that the mitochondria depolarization through the application of FCCP promotes Ca^2+^ waves (Zhao et al., 2013). Furthermore, the effects of FCCP were counteracted by the application of mPTP blocker cyclosporine A (Zhao et al., 2013). Also, our previous experimental observations using cyclophilin D knockout mouse model showed attenuation of Ca^2+^ waves (Gordan et al., 2016). Elevation of cytosolic ROS during mPTP opening could be a significant factor, and experimental evidence showed that oxidative stress during mitochondrial depolarization slightly alters SR Ca^2+^ leaks (Ca^2+^ spark) amplitude but drastically increases its frequency (Yan et al., 2008; Zhou et al., 2011; Williams et al., 2013). Furthermore, ROS can oxidize CaMKII and enhance its activation. It has been shown that ROS and CaMKII activation act on the major Ca^2+^ handling proteins, such as RyRs and SERCA (Hund and Rudy, 2004; Wehrens et al., 2004; Ai et al., 2005; Guo et al., 2006; Zima and Blatter, 2006; Wagner et al., 2013). The direct redox effect of ROS increases the leakiness of RyRs and decreases the strength of SERCA (Zima and Blatter, 2006; Wagner et al., 2013), whereas CaMKII activation increases both the leakiness of RyRs and the strength of SERCA (Hund and Rudy, 2004; Ai et al., 2005; Mattiazzi and Kranias, 2014). Thus, the observed Ca^2+^ waves in experiments during mitochondrial depolarization are the consequences of the combined effects of the above factors. However, what is the primary player remains unclear. Here, by using our previously established physiologically detailed computer model, we show that our simulation results agree with the experimental observations that mitochondrial depolarization could induce spontaneous Ca^2+^ release and DADs. Furthermore, we found that it is the redox effect on RyRs that causes the DADs under the acute effect of mitochondrial depolarization, and the redox effect of ROS on reducing SERCA strength may not be sufficient to suppress DADs (Figure 2).

In addition, we indeed observed an increase in CaMKII activation due to the mPTP opening (Figure 1B), but the dynamics of CaMKII activation appeared much slower than that of the ROS. The CaMKII activation was increased from ∼0.2% under control to ∼0.6% during the mitochondrial depolarization for a 45-sec long simulation (Figure 1B). A further simulation showed that CaMKII activation could reach up to ∼40% for a much more extended duration (1200 s). These results suggest that CaMKII activation may be too slow to play an essential role in inducing DADs under the acute effect of mitochondrial depolarization. However, simulations with different clamped CaMKII activation levels reveal that CaMKII activation may play a vital role in causing spontaneous Ca^2+^ release and DADs chronically, since a higher CaMKII activation level caused a greater DAD amplitude (Figure 4A).

Similarly, we have observed a slight change in cytosolic ATP under the acute effect of mitochondrial depolarizations in our simulations (Figure 1B). Therefore, within a relatively short duration after mitochondrial depolarization, ATP may not be involved in the genesis of spontaneous Ca^2+^ release and DADs. Clamped ATP simulations showed that a lower cytosolic ATP level is linked to a smaller amplitude of DADs (Figure 4B). This is because the reduction of ATP impaired SERCA activity and suppress the DADs, which agree well with experimental evidence that ATP synthase inhibitor, oligomycin, does not promote DADs (Zhao et al., 2013). Although ATP reduction seems to suppress Ca^2+^ waves and DADs, our simulations and other’s experimental work suggest that ATP reduction could promote Ca^2+^ alternans, which is still arrhythmogenic (Hüser et al., 2000; Kockskämper et al., 2005; Zima and Blatter, 2006; Pandey et al., 2021).

### Mitochondrial Ca^2+^ Uniporter Overexpression and Delayed Afterdepolarizations

Mitochondrial Ca^2+^ uptake has been reported to rise in heart failure (Santulli et al., 2015; Xie et al., 2018) and can promote EADs (Xie et al., 2018) and Ca^2+^ alternans (Pandey et al., 2021). Our previous work demonstrated MCU upregulation could promote EADs in heart failure conditions without mPTP openings (Xie et al., 2018). And MCU upregulation promotes Ca^2+^ alternans through the Ca^2+^ dependent opening of mPTPs (Pandey et al., 2021). The previous experiment by Zhao et al. (2013) reported that the mitochondrial Ca^2+^ efflux in the proximity of the junctional SR played an essential role in the regulation of Ca^2+^ waves. Furthermore, our previous study has shown that MCU overexpression can lead to Ca^2+^ overload in mitochondria (Song et al., 2019). Also, there is evidence that mitochondrial Ca^2+^ overload can cause the Ca^2+^-dependent opening of mPTP (Kwong and Molkentin, 2015), resulting in mitochondrial depolarization (Santulli et al., 2015). Here, our simulation study shows that increasing MCU activity promotes spontaneous Ca^2+^ release and DADs (Figure 3). The underlying mechanism revealed in our simulations is that increasing MCU activity enhanced mitochondrial depolarization through the Ca^2+^-dependent openings of mPTP, which resulted in spontaneous Ca^2+^ release primarily due to the direct redox effect of ROS on RyRs.

### Pathophysiological and Clinical Relevance

Mitochondrial dysfunction has been associated with increased arrhythmic risk (Santulli et al., 2015; Shimizu et al., 2015; Xie et al., 2015, 2018; Gordan et al., 2016), which could account for mortality in many cardiac diseases such as cardiomyopathy, heart failure, and ischemia/reperfusion injury (IRI). We have demonstrated that the direct redox effect of ROS on RyRs plays a critical role in promoting Ca^2+^ waves and DADs under the acute effect of mitochondrial depolarization. Furthermore, the upregulation of MCU can promote DADs through Ca^2+^-dependent opening of mPTPs. These findings suggest that pharmacological interventions targeted at avoiding ROS buildup and MCU upregulation may provide novel therapeutics to prevent or treat cardiac arrhythmias.

### Limitations

This detailed model coupled AP, CRUs, and mitochondria to capture excitation-contraction-metabolism coupling in ventricular myocytes. However, it has some limitations. For instance, heterogeneities in T-tubule networks and distributions of ion channels and Ca^2+^ handling proteins are a few examples (Soeller and Cannell, 1999; Baddeley et al., 2009) that should be considered in the future analysis. These heterogeneities in T-tubule networks may alter the genesis of DADs (Song et al., 2018).

We note that in Figure 3A, for α_*mPTP*_=1, there was virtually no DAD occurring even with α_*MCU*_=50, suggesting that the proposed mechanism of increasing MCU activity inducing DADs in this study requires a certain basal level of mPTP opening. In this model, we consider the mPTP gating kinetics only mitochondrial Ca^2+^ dependent. However, the ROS-induced ROS release mechanism also impacts the mPTP open probability (Zorov et al., 2000, 2006), which is essential for modeling mitochondrial depolarization waves (Yang et al., 2010; Zhou et al., 2010; Nivala et al., 2011). Thus, this ROS-induced ROS release mechanism may provide another critical positive feedback loop between mitochondrial and cytosolic Ca^2+^ instability. In the future, the ROS-induced ROS release will be added to our model to study the role of mitochondrial depolarization waves in the genesis of arrhythmias in diseased conditions, such as heart failure.

## Data Availability Statement

The original contributions presented in the study are included in the article/supplementary material, further inquiries can be directed to the corresponding author/s.

## Author Contributions

VP performed the simulations, analyzed simulation results, and drafted the manuscript. ZS prepared the figures. All the authors conceived and designed the study, interpreted the results, and edited and revised the manuscript.

## Conflict of Interest

The authors declare that the research was conducted in the absence of any commercial or financial relationships that could be construed as a potential conflict of interest.

## Publisher’s Note

All claims expressed in this article are solely those of the authors and do not necessarily represent those of their affiliated organizations, or those of the publisher, the editors and the reviewers. Any product that may be evaluated in this article, or claim that may be made by its manufacturer, is not guaranteed or endorsed by the publisher.
